# Weighted multi-scale and wavelet-enhanced Segment Anything Model for salient object detection

**DOI:** 10.1371/journal.pone.0355742

**Published:** 2026-08-11

**Authors:** Zhe Liu, Dan Tian

**Affiliations:** School of Intelligent Science and Information Engineering, Shenyang University, Shenyang, Liaoning, China; University of Electronic Science and Technology of China, CHINA

## Abstract

Salient Object Detection (SOD) is concerned with isolating the visually most noticeable objects in an image via precise segmentation. Previous approaches, especially those that adapt the Segment Anything Model (SAM), often yield saliency maps that contain incomplete object masks, blurred boundaries, and a lack of fine-grained details. This issue is especially severe for scenes with objects of different scales or complex textures. We argue that these issues stem from three inherent limitations of existing adaptation strategies: (1) existing adapters rely on rigid multi-scale fusion strategies, lacking learnable cross-scale calibration to handle objects of diverse sizes, (2) simple feature concatenation ignores cross-level semantic correlations, (3) spatial-domain operations inevitably discard high-frequency details. The proposed WMW-SAM, a Weighted Multi-Scale and Wavelet-Enhanced SAM, is designed to handle these limitations for SOD. Specifically, we develop the Weighted Multi-Scale Adapter (WMSA), which utilizes learnable weighting across multiple receptive fields to calibrate features of different scales. Then, our Multi-level Feature Cross-fusion Module (MFCM) employs cascaded top-down cross-attention to facilitate deep interaction that connects high-level semantics with low-level details. Finally, we develop a Detail Enhancement Module (DEM) that leverages the Discrete Wavelet Transform (DWT) to explicitly extract and enhance high-frequency sub-bands. This operation effectively recovers sharp boundaries and intricate textures, which are often overlooked by spatial-domain operations. Extensive experiments on multiple benchmark datasets demonstrate the superior performance of our proposed WMW-SAM, which achieves accurate and detailed saliency predictions.

## 1. Introduction

Salient Object Detection (SOD) [1] aims to identify the most visually salient objects or regions in an image. As a fundamental vision task, it serves as a critical step for numerous downstream applications, including object tracking [2,3], scene segmentation [4,5], and person re-identification [6,7]. Moreover, techniques developed for SOD have also been adapted to related tasks, such as multi-scale synthetic aperture radar (SAR) object detection [8] and energy-efficient water-surface object detection [9]. The past decade has witnessed remarkable success of methods based on Convolutional Neural Networks (CNNs) for SOD. Nevertheless, the limited receptive fields of CNNs hinder their ability to capture global contextual information. Notably, thanks to the global perception capability enabled by the self-attention mechanism, Vision Transformers (ViT) [10] achieve performance improvements in SOD. However, for complex scenes, the performance and generalization ability of these SOD models remain low. This is largely due to limited training data and large domain gaps.

The Segment Anything Model (SAM) [11] is recognized as a powerful visual foundation model for universal image segmentation. It leverages over one billion training masks and demonstrates exceptional generalization on many segmentation tasks [12–16]. However, its performance depends heavily on manual prompts for object identification, such as specific points, bounding boxes, or coarse masks [17]. In the context of SOD, it is impractical to provide such prompts, as ground truth is unavailable during inference. SAM can automatically generate masks via grid prompts, but these masks are class-agnostic and cannot separate salient objects from the background. Moreover, full fine-tuning of SAM for SOD is computationally expensive and often leads to performance degradation or catastrophic forgetting of its pre-trained knowledge.

**Fig 1:**
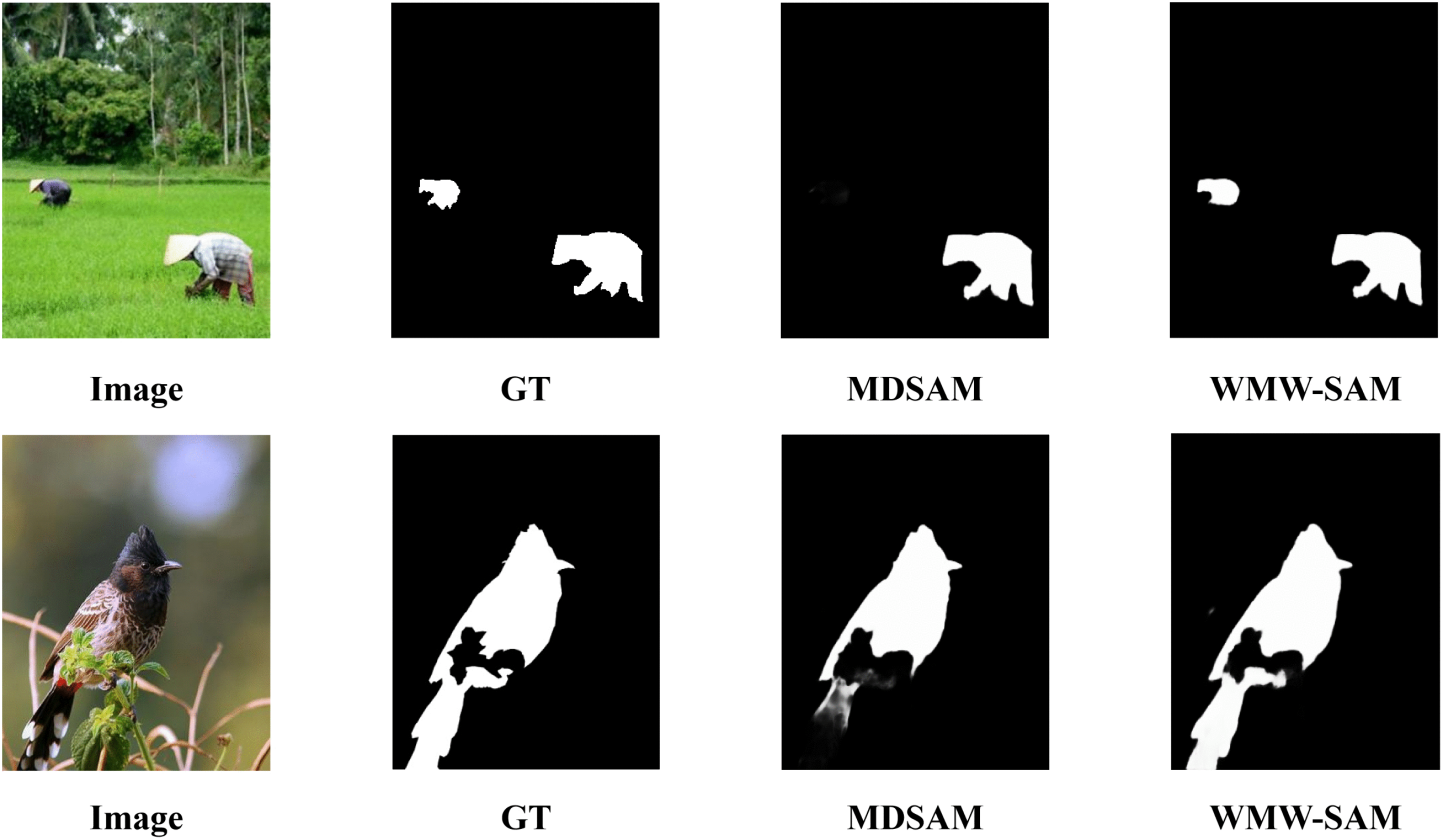
Visual examples of objects with diverse scales and intricate details. The predictions are generated by MDSAM [17] and WMW-SAM methods.

In order to address these issues, recent works employ Parameter-Efficient Fine-Tuning (PEFT) for the adaptation of foundation models to different tasks. Specifically, existing methods primarily focus on multi-scale modeling and feature fusion to transfer SAM to the SOD task. Nevertheless, these approaches exhibit inherent limitations. First, existing fusion strategies for multi-scale features lack the learnable weighting mechanism required to handle objects of different sizes. Second, simple fusion strategies fail to capture intricate cross-level semantic correlations. Finally, as detail enhancement remains restricted to the spatial-domain, these methods are prone to losing high-frequency structural information. Consequently, as shown in Fig 1, current methods still suffer from incomplete object masks and blurred boundaries in complex scenes.

To overcome these limitations, we propose the Weighted Multi-Scale and Wavelet-Enhanced SAM (WMW-SAM), a novel framework for SOD. Our approach effectively adapts SAM to the SOD task by incorporating multi-scale perception and frequency-domain detail enhancement. Specifically, we introduce the Weighted Multi-Scale Adapter (WMSA), which employs a learnable weighting mechanism across multiple receptive fields to calibrate features of different scales. Furthermore, we introduce a Multi-level Feature Cross-fusion Module (MFCM) based on Multi-level Cross-Attention (MCA). MFCM uses cascaded top-down interaction rather than simple concatenation to effectively fuse high-level semantics with low-level details. Finally, we develop a Detail Enhancement Module (DEM) that leverages the Discrete Wavelet Transform (DWT) to explicitly extract and enhance high-frequency sub-bands. This frequency-aware mechanism successfully recovers sharp boundaries and complex textures that are often lost in spatial-domain operations. Extensive experiments on multiple SOD benchmark datasets demonstrate the superior performance of our WMW-SAM over existing methods.

Our main contributions are summarized as follows:

- We design a Weighted Multi-Scale Adapter (WMSA) to address the rigid multi-scale feature aggregation in existing SAM-based SOD methods. This adapter employs learnable weighting across multiple receptive fields and calibrates multi-scale features for salient objects of diverse sizes.
- We propose a Multi-level Feature Cross-fusion Module (MFCM) to overcome the ineffective cross-level semantic fusion caused by simple concatenation. The MFCM uses cascaded top-down cross-attention to facilitate deep interaction that connects high-level semantics with low-level details.
- We further develop a Detail Enhancement Module (DEM) to recover high-frequency structural details that spatial-domain operations inevitably lose. The DEM leverages the Discrete Wavelet Transform (DWT) to explicitly extract and enhance high-frequency sub-bands, restoring sharp boundaries and intricate textures.
- We conduct extensive experiments on multiple SOD benchmark datasets to verify our method’s superiority and generalization capability.

## 2. Related work

### 2.1. Salient object detection

Current mainstream SOD methods can be roughly divided into two categories: CNN-based and Transformer-based [17]. The CNN-based methods typically use deep convolutional networks such as VGGNet [18] and ResNet [19] as backbones to extract multi-scale features and then generate saliency maps through decoder structures. For example, Zhang et al. [20] introduce a recursive learning framework for SOD, which adaptively combines multi-resolution features and incorporates edge-aware information to refine object boundaries. Zeng et al. [21] utilize a global-local refinement framework to solve the problem of detail loss and boundary inaccuracy in high-resolution SOD. Wei et al. [22] employ a selective feature fusion module and a cascaded feedback decoder to address the discrepancies between multi-level features and enhance the prediction of fine details. Mohammadi et al. [23] design a content-aware guidance mechanism that integrates low-level and high-level features to suppress non-salient regions resembling salient objects while handling salient objects with non-uniform appearance. Liu et al. [24] propose a unified framework with dynamic feature integration and task-adaptive attention to simultaneously solve salient object, edge, and skeleton detection and reduce task conflicts. Zhao et al. [25] adopt the gate mechanism to balance the contribution of each encoder block and reduce non-salient information. Pang et al. [26] introduce a multi-scale interactive network to improve spatial coherence across levels. Wei et al. [27] propose a label decoupling framework that decomposes the saliency label into body and detail maps to solve the problem of prediction difficulty and pixel imbalance near object boundaries. Wang et al. [28] design a multiple enhancement network that uses pixel, region, and object level supervision. It produces clearer boundaries and more complete salient objects in complex scenes. This network uses multi-level hybrid losses and multiscale feature enhancement to refine boundaries of complex objects in cluttered scenes. Although these CNN-based methods make significant progress, they often produce unsatisfactory results in complex situations. The main reason is CNNs’ inherent local receptive fields, which severely limit the capture of the global semantic context critical for SOD.

Recently, thanks to the global perception capability of the self-attention mechanism, ViT [10] has demonstrated excellent performance in SOD. Based on these advantages, Liu et al. [29] leverage T2T-ViT [30] to capture long-range dependencies and integrate multi-level features to boost detection performance. To further optimize saliency features, Yun et al. [31] design a self-refined network with pyramid Transformers, which enhances both global semantics and local details. Zhuge et al. [32] adopt the Swin Transformer [33] to extract multi-scale features, thereby improving the structural integrity of salient regions. Wang et al. [34] utilize a combination of multiple Transformers to learn robust local-global representations in the scribble-based RGB-D SOD task. Moreover, Deng et al. [35] propose a recurrent multi-scale architecture to address the challenge of generating high-quality saliency maps for high-resolution images. Although such Transformer-based models achieve impressive performance, they often struggle to capture fine-grained structural details. More critically, compared with large-scale visual foundation models, they still have limited generalization ability in complex and unseen scenes. Our approach exploits the excellent feature extraction and generalization provided by vision foundation models, and applies them to the SOD task to obtain improved results.

### 2.2. Segment anything model

SAM [11] is a groundbreaking visual foundation model for universal image segmentation. Through appropriate adjustments, it demonstrates remarkable performance on various downstream tasks [12–16]. Nevertheless, original SAM heavily relies on manual prompts, which are unavailable in fully automatic SOD. Although full fine-tuning can transfer SAM to SOD, it inevitably introduces too many trainable parameters and often leads to performance degradation due to catastrophic forgetting [17]. To mitigate this issue, PEFT methods have been widely explored. For instance, Cui et al. [36] efficiently fine-tune SAM for SOD with a Low Rank Adaptation (LoRA). Xu et al. [37] introduce a SAM-based multidimensional exploration method for weakly supervised SOD. Ke et al. [38] enhance SAM by introducing a learnable high-quality output token into its mask decoder to generate more accurate and detailed segmentation masks. Although effective in certain cases, these methods still fail to fully capture multi-scale contextual information required for SOD.

To solve this problem, Gao et al. [17] introduce a Lightweight Multi-Scale Adapter (LMSA) to inject multi-scale features into the SAM encoder. However, this approach relies on rigid multi-scale aggregation and lacks learnable cross-scale calibration to handle diverse object scales. Furthermore, the simple fusion mechanisms and spatial-domain decoding strategies in these methods cannot preserve fine-grained edge details and therefore fail to segment complex scenes accurately. To overcome these issues, we leverage a parameter-efficient WMSA module to adapt SAM for the SOD task. Additionally, we introduce lightweight modules to exploit multi-level interactions and frequency-domain details, and obtain superior results on the SOD task.

## 3. Our proposed method

**Fig 2:**
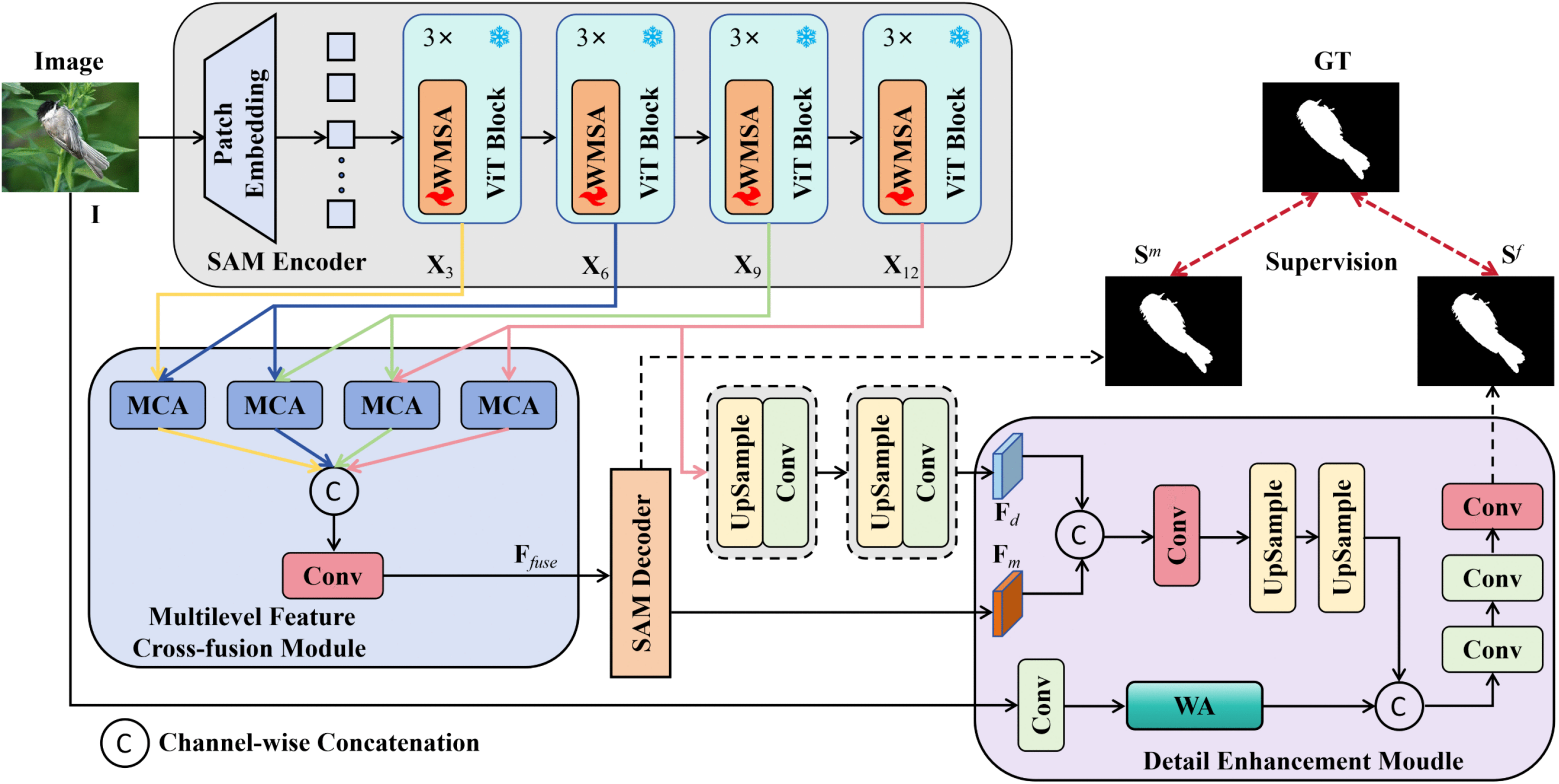
Architecture of our WMW-SAM. The frozen SAM encoder incorporates the Weighted Multi-Scale Adapter (WMSA) for multi-scale perception. Then, the extracted hierarchical features (**X**_3_, **X**_6_, **X**_9_, **X**_12_) are integrated through the Multi-level Feature Cross-fusion Module (MFCM), which employs Multi-level Cross-Attention (MCA). Finally, the Detail Enhancement Module (DEM) refines the initial prediction using Wavelet Attention (WA) to recover high-frequency edge details.

This section presents the Weighted Multi-Scale and Wavelet-Enhanced SAM (WMW-SAM) to address the limitations of existing strategies that adapt SAM to SOD. As shown in Fig 2, WMW-SAM integrates three core components: the Weighted Multi-Scale Adapter (WMSA), the Multi-level Feature Cross-fusion Module (MFCM), and the Detail Enhancement Module (DEM). The following subsections present the specific formulations of these modules.

### 3.1. Weighted multi-scale adapter

While SAM demonstrates good performance across many segmentation tasks, its dependence on manual prompts and lack of task-specific multi-scale features hinder its direct use in automatic SOD. Full fine-tuning can adapt SAM to SOD, but it incurs prohibitive computational cost and often overfits on limited data. Consequently, parameter-efficient adaptation via lightweight adapters [39] has become the dominant approach. A recent study by Gao et al. [17] introduces an LMSA module that injects multi-scale information into SAM to capture finer spatial details. However, this approach relies on rigid multi-scale aggregation and lacks learnable cross-scale calibration to handle salient objects with diverse sizes and shapes. To overcome this, we introduce a WMSA module. It employs parallel depth-wise convolutions (e.g., 3 × 3, 5 × 5, 7 × 7) to construct multi-scale receptive fields and fuses features of different scales via global learnable scalar weights. By embedding WMSA into the SAM encoder, our WMW-SAM acquires robust multi-scale perception with only a few extra parameters. This design maintains high training efficiency and preserves the foundation model’s generalization ability under complex scenes.

**Fig 3:**
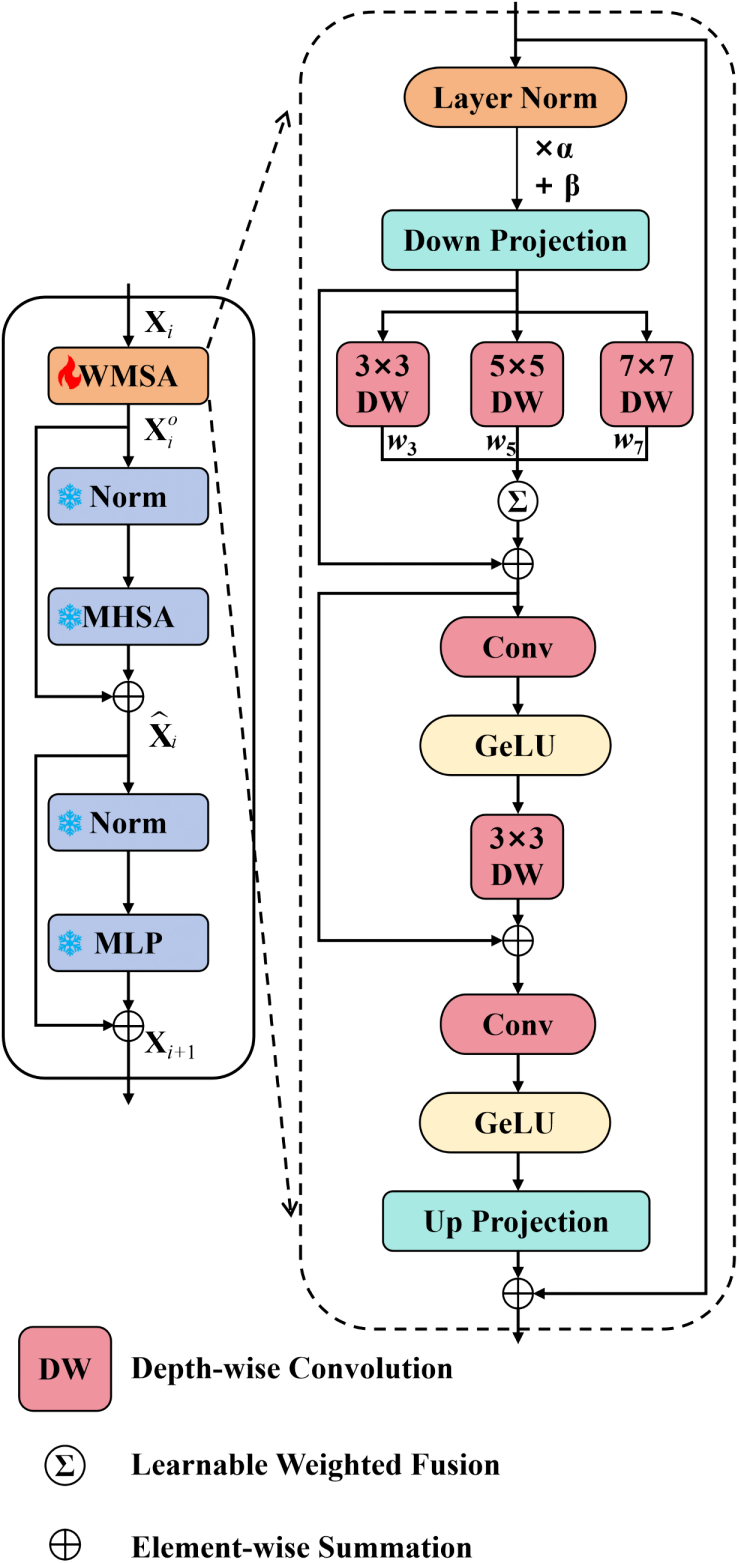
Illustration of the proposed WMSA. We add WMSA before the first normalization in each Transformer block.

As shown in Fig 3, each Transformer block of the SAM encoder comprises a Multi-Head Self-Attention (MHSA) [40], a Multi-Layer Perceptron (MLP), and Layer Normalization [41]. Specifically, given the input feature, the operations in the *i*-th block are formulated as:


$$\begin{array}{c} {\hat{𝐗}}_{i}=MHSA\left(LN\left({𝐗}_{i}\right)\right)+{𝐗}_{i}, \end{array}$$ (1)

$$\begin{array}{c} {𝐗}_{i+1}=MLP\left(LN\left({\hat{𝐗}}_{i}\right)\right)+{\hat{𝐗}}_{i}, \end{array}$$ (2)

where ${𝐗}_{i}$ denotes an input to the *i*-th Transformer block, ${\hat{𝐗}}_{i}$ represents the intermediate output, and *LN* corresponds to Layer Normalization. To adapt SAM for SOD, we utilize WMSA before the first *LN* in each Transformer block.

The architecture of WMSA is illustrated in Fig 3. Specifically, we first calibrate the distribution of the input feature ${𝐗}_{i}$, then project it into a low-dimensional subspace via a learnable linear layer, thereby reducing computational complexity:


$$\begin{array}{c} {𝐗}_{d}=D_{i}(\alpha \cdot LN({𝐗}_{i})+\beta ), \end{array}$$ (3)

where *α* and *β* are learnable scale and shift parameters, respectively, and *D*_*i*_ denotes the linear projection layer for dimensionality reduction in the *i*-th adapter.

Subsequently, we introduce a weighted fusion mechanism that enhances model sensitivity to salient objects with various sizes. It consists of three parallel depth-wise convolutional branches with kernel sizes of 3 × 3, 5 × 5, 7 × 7, which capture spatial information at different receptive fields. The resulting multi-scale features are combined using global learnable weighting coefficients *w*_*k*_. With an internal residual connection, this fusion process is formulated as:


$$\begin{array}{c} {𝐅}_{ms}={𝐗}_{d}+\frac{\sum_{k\in \left\{3,5,7\right\}}w_{k}\cdot DWConv_{k}\left({𝐗}_{d}\right)}{\sum_{k\in \left\{3,5,7\right\}}w_{k}+\varepsilon }, \end{array}$$ (4)

where *DWConv*_*k*_ denotes the depth-wise convolutional layer with a k × k kernel, and *ε* is a small constant for numerical stability. To apply the depth-wise convolutions, the projected 1D token sequence ${𝐗}_{d}\in {\mathbb{R}}^{B\times N\times C^{\prime }}$ (where *N* = *H* × *W* and $C^{\prime }$ denotes the reduced channel dimension) is first reshaped into a 2D spatial grid ${\mathbb{R}}^{B\times C^{\prime }\times H\times W}$. After the parallel convolutions, the features are flattened back to the sequence format. Since the SAM encoder adopts a standard ViT architecture without hierarchical down-sampling, the spatial resolution *H* × *W* remains consistent across all stages. Furthermore, the original absolute positional embeddings, added before the first block, are continuously propagated via residual connections. Our WMSA operates exclusively on the reshaped grids without modifying or recalibrating these embeddings. The depth-wise convolutions operate on the 2D spatial grids and thereby naturally preserve the structural ordering. Consequently, the local features extracted by our WMSA are spatially consistent with the tokens processed by the subsequent global self-attention layers. To further improve the fused features, we introduce a lightweight residual block to refine the multi-scale representation:


$$\begin{array}{c} {𝐅}_{ref}={𝐅}_{ms}+DWConv_{3\times 3}\left(\sigma \left(Conv_{1\times 1}\left({𝐅}_{ms}\right)\right)\right), \end{array}$$ (5)

where *σ* denotes the GELU activation function [42]. Subsequently, we apply a 1 × 1 convolution to compress and integrate the refined features, and then use GELU for nonlinear activation. The output is projected back to the original dimensionality via another linear layer. Finally, a residual connection yields the output of the WMSA module:


$$\begin{array}{c} {𝐗}_{i}^{o}=U_{i}\left(\sigma \left(Conv_{1\times 1}\left({𝐅}_{ref}\right)\right)\right)+{𝐗}_{i}, \end{array}$$ (6)

where *U*_*i*_ denotes the linear up-projection layer of the *i*-th adapter.

In summary, with the proposed WMSA, our framework adapts SAM to the SOD task with only a few additional parameters. Moreover, unlike static adapters with fixed multi-scale aggregation, our method enables learnable recalibration of multi-scale features, leading to more accurate identification and localization of salient objects in complex scenes.

### 3.2. Multi-level feature cross-fusion module

The SAM encoder produces features at multiple levels. Shallow layers retain fine spatial details, while deeper layers capture high-level semantics. However, for SOD in complex scenes, sole reliance on high-level features from deep layers may fail to accurately detect objects. Therefore, effective fusion of features from different levels becomes necessary. The original SAM uses only the final output for mask decoding and discards valuable information from intermediate layers. Basic fusion methods like concatenation or summation do not fully explore the connections between layers. To overcome these issues, we propose the MFCM module. Based on MCA, MFCM integrates features from multiple stages of the SAM encoder in a more comprehensive manner.

**Fig 4:**
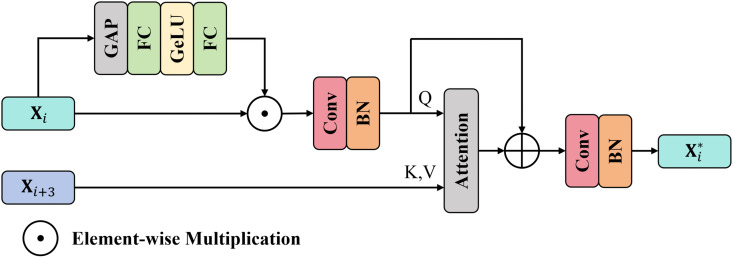
Illustration of the MCA module.

The MCA module uses high-level semantic context to refine low-level features. It builds a top-down pathway that helps shallow layers suppress background noise and highlight fine details related to salient objects. As shown in Fig 4, we first apply channel attention [43] to recalibrate the input features and improve their representational quality. Next, we employ cross-attention with the current low-level features as the Query and the higher-level features as the Key and Value. This design enables shallow features to query and integrate relevant high-level semantics, which leads to better cross-level interaction and semantic alignment. This process is summarized as:


$$\begin{array}{c} {𝐗}_{i}^{\text{'}}=Conv_{1\times 1}({𝐗}_{i}⊙\varphi \left(\sigma \left(\varphi \left(GAP\left({𝐗}_{i}\right)\right)\right)\right), \end{array}$$ (7)

$$\begin{array}{c} Q=\varphi \left({𝐗}_{i}^{\text{'}}\right),K=\varphi \left({𝐗}_{i+3}\right),V=\varphi \left({𝐗}_{i+3}\right), \end{array}$$ (8)

$$\begin{array}{c} Attention\left(Q,K,V\right)=Softmax(Q⊗K^{T})⊗V, \end{array}$$ (9)

$$\begin{array}{c} {𝐗}_{i}^{*}=BN\left(Conv_{1\times 1}\left({𝐗}_{i}^{\text{'}}+Attention\left(Q,K,V\right)\right)\right), \end{array}$$ (10)

In the above formulas, *φ* corresponds to linear projection, and *BN* to batch normalization. ${𝐗}_{i}^{{\;}^{\prime }}$ is the intermediate feature obtained after channel attention and 1 × 1 convolution. For the deepest feature ${𝐗}_{\text{12}}$, no higher-level semantic reference exists. Therefore, it skips the cross-attention stage and is refined only by the channel attention module.

The MFCM refines features in a top-down cascade manner based on MCA. In this process, each shallow feature receives semantic guidance from deeper features. Then, a 1 × 1 convolution processes the concatenation of all refined features (${𝐗}_{\text{3}}^{*},\;{𝐗}_{\text{6}}^{*},\;{𝐗}_{\text{9}}^{*},\;{𝐗}_{\text{12}}^{*}$) and produces ${𝐅}_{fuse}$, which supplies multi-level information to the mask decoder:


$$\begin{array}{c} {𝐗}_{\text{12}}^{*}=MCA\left({𝐗}_{12}\right), \end{array}$$ (11)

$$\begin{array}{c} {𝐗}_{i}^{*}=MCA\left({𝐗}_{i},{𝐗}_{i+3}\right),i=3,6,9 \end{array}$$ (12)

$$\begin{array}{c} {𝐅}_{fuse}=Conv_{1\times 1}\left(Concat\left({𝐗}_{\text{3}}^{*},{𝐗}_{\text{6}}^{*},{𝐗}_{\text{9}}^{*},{𝐗}_{\text{12}}^{*}\right)\right), \end{array}$$ (13)

Unlike the original SAM, which uses only the final encoder output, our MFCM fuses features from multiple levels of the SAM encoder, allowing the decoder to produce clearer saliency maps.

### 3.3. Detail enhancement module

Although WMSA and MFCM adapt SAM to SOD and fuse multi-level features, the patch embedding in the SAM encoder still loses fine spatial details. Moreover, simple upsampling in the SAM decoder cannot fully recover high-frequency information. This often leads to poor detection of objects with complex edges or slender shapes. To overcome this, we propose a DEM module that uses frequency-domain information to restore lost structural details.

**Fig 5:**
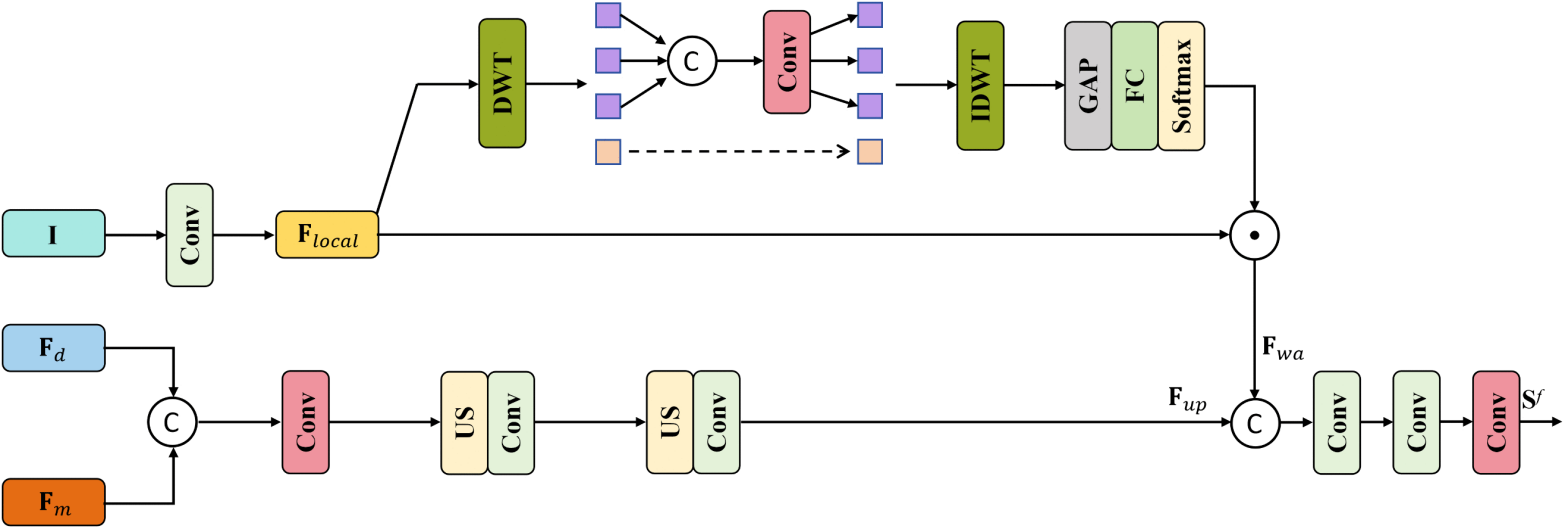
Illustration of the proposed DEM.

As shown in Fig 5, DEM consists of a primary decoding branch and an auxiliary detail branch. The primary branch gradually resamples features from the mask decoder up to the input resolution, while fine details from the original image are extracted by an auxiliary branch to support the primary feature. In the auxiliary branch, we introduce Wavelet Attention (WA), which differs from standard spatial-domain edge extraction. The wavelet transform [44] is used to isolate and enhance high-frequency components in the frequency-domain. This enables precise recovery of edge details.

The primary branch reconstructs the spatial resolution of the final prediction. First, we concatenate the high-level semantic feature ${𝐅}_{m}$ from the mask decoder with the upsampled feature ${𝐅}_{d}$ from the final layer of the SAM encoder, and we compress its channel dimension via a 1 × 1 convolution. Then, the combined feature enters multiple upsampling stages. Each stage consists of bilinear interpolation followed by a 3 × 3 convolution. This process restores the feature maps to the original input resolution, formulated as follows:


$$\begin{array}{c} {𝐅}_{c}=Conv_{1\times 1}\left(Concat\left({𝐅}_{m},{𝐅}_{d}\right)\right), \end{array}$$ (14)

$$\begin{array}{c} {𝐅}_{up}=Conv_{3\times 3}\left(US_{\times 2}\left(Conv_{3\times 3}\left(US_{\times 2}\left({𝐅}_{c}\right)\right)\right)\right), \end{array}$$ (15)

where *US*_×2_ represents a twofold upsampling via bilinear interpolation.

The upsampled feature ${𝐅}_{up}$ provides a coarse feature, which exhibits blurred boundaries and lacks high-frequency details due to the smoothing effect of repeated bilinear interpolation. To address this, we employ WA in the auxiliary branch to recover edge information in the frequency-domain. First, we use a 3 × 3 convolution on the input image **I** to extract local features:


$$\begin{array}{c} {𝐅}_{local}=Conv_{3\times 3}\left(𝐈\right), \end{array}$$ (16)

Subsequently, **F**_*local*_ is fed into the WA module. This module first applies a single-level Haar DWT to decompose the feature into one low-frequency sub-band and three high-frequency sub-bands. The three high-frequency sub-bands correspond to structural details in the vertical, horizontal, and diagonal directions:


$$\begin{array}{c} \left(𝐋,{𝐇}_{v},{𝐇}_{h},{𝐇}_{d}\right)=DWT\left({𝐅}_{local}\right), \end{array}$$ (17)

We apply learnable thresholds to these high-frequency sub-bands. Then, the results are concatenated and fused by a 1 × 1 convolution. Finally, they are combined with the low-frequency sub-band and reconstructed via the Inverse Discrete Wavelet Transform (IDWT) into an edge-enhanced feature:


$$\begin{array}{c} {𝐅}_{e}=IDWT\left(Conv_{1\times 1}\text{(}Concat\left({𝐇}_{v},{𝐇}_{h},{𝐇}_{d}\right)\right),𝐋), \end{array}$$ (18)

To generate attention weights, we first apply Global Average Pooling (GAP) to aggregate spatial information, and then use Flatten to expand the feature. Then, wavelet attention-guided weights are generated via the Softmax function. These weights multiply the original input feature to yield the final frequency-domain enhanced detail feature:


$$\begin{array}{c} {𝐅}_{wa}=Softmax\left(Flatten\left(GAP\left({𝐅}_{e}\right)\right)\right)⊙{𝐅}_{local}, \end{array}$$ (19)

Finally, we concatenate the detail-rich feature **F**_*wa*_ with the primary feature **F**_*up*_, and output the final saliency prediction through a series of convolutions:


$$\begin{array}{c} {𝐒}^{f}=Conv_{1\times 1}\left(Conv_{3\times 3}\left(Conv_{3\times 3}\left(Concat\left({𝐅}_{wa},{𝐅}_{up}\right)\right)\right)\right), \end{array}$$ (20)

In summary, DEM combines the semantic feature from the primary branch with the frequency-enhanced detail from the auxiliary branch. This helps recover the structural information lost in the original SAM. As a result, our WMW-SAM produces more accurate saliency maps.

### 3.4. Loss functions

During training, we combine *BCE* loss, *IoU* loss, and *L*1 loss to optimize the parameters. Both the final refined prediction **S**^*f*^ and the intermediate coarse prediction **S**^*m*^ are used for loss computation to better guide the intermediate layers and facilitate gradient propagation. The total loss is the sum of these two parts:


$$\begin{array}{c} ℒ\left(𝐒,𝐆\right)={ℒ}_{BCE}+{ℒ}_{IoU}+{ℒ}_{L1}, \end{array}$$ (21)

$$\begin{array}{c} {ℒ}_{total}={ℒ}_{f}\left({𝐒}^{f},𝐆\right)+{ℒ}_{m}\left({𝐒}^{m},𝐆\right), \end{array}$$ (22)

where **G** denotes the salient object’s ground truth map.

## 4. Experiments

### 4.1. Experiment settings

**Datasets.** We train our network on the DUTS-TR [45] dataset, which contains 10,553 images. We evaluate our method on five widely-used benchmark datasets: DUTS-TE [45] (5,019 images), DUT-OMRON [46] (5,168 images), HKU-IS [47] (4,447 images), ECSSD [48] (1,000 images), and PASCAL-S [49] (850 images).

**Metrics.** To quantitatively assess the performance of our WMW-SAM, we adopt four metrics commonly used in SOD research: Mean Absolute Error (*MAE*) [50], maximum F-measure ($F_{\beta }^{max}$) [45], S-measure ($S_{m}$) [51], and mean Enhanced-alignment Measure (*E*_*m*_) [52].

**Implementation Details.** Our model is implemented in PyTorch and trained on a single NVIDIA GeForce RTX 4090 GPU. We initialize the image encoder and mask decoder with pretrained weights from the SAM1 using the ViT-B backbone, and randomly initialize all new modules in WMW-SAM. Input images are resized to 384 × 384, and the batch size is set to 16. We utilize the AdamW optimizer with a weight decay of 1e-4. During training, the SAM encoder remains frozen. The learning rates for other pretrained weights and the newly introduced modules are set to 3e-5 and 3e-4, respectively. The entire training process lasts for 100 epochs, including a warm-up period of 5 epochs.

### 4.2. Comparative experiments

To comprehensively evaluate the effectiveness of our WMW-SAM, we compare it with 13 representative methods, including DFI [24], MINet [26], F3Net [22], LDF [27], GateNet [25], CAGNet [23], VST [30], ICON [33], SelfReformer [32], MENet [33], BRRF [53], SAM [11], and MDSAM [17].

**Fig 6:**
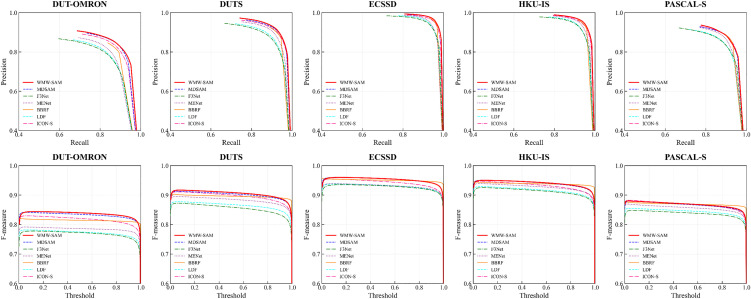
Precision-recall and F-measure curves of the proposed method and other methods on five SOD datasets.

**Quantitative Evaluation.** Tables 1 and 2 show the quantitative results on five widely used benchmark datasets. We compare our method with 13 other methods in terms of *MAE*, $F_{\beta }^{max}$, $S_{m}$, and *E*_*m*_. The results show that our method achieves the best performance on DUTS, HKU-IS, and ECSSD and remains highly competitive on DUT-OMRON. Notably, our method achieves convincing results on the $F_{\beta }^{max}$ metric across five datasets compared to previous models. Although our WMW-SAM performs slightly worse on the PASCAL-S dataset, it still achieves the best overall results. Compared to the original SAM, our model shows clear improvement with only a few additional parameters, even at a lower input resolution. Our core modules WMSA, MFCM, and DEM introduce merely 1.38M, 1.61M, and 0.23M trainable parameters, respectively. Including other auxiliary components (e.g., dimension matching layers and the final output convolution), the complete WMW-SAM totals 95.52M, which adds an overhead of approximately 5.58M to the frozen SAM. Compared with MDSAM, our approach delivers better accuracy with fewer parameters and runs at 62 FPS. In addition, we present the precision-recall [54] and F-measure curves [55] in Fig 6.

**Table 1:** Quantitative comparison of our method with other methods on DUTS-TE and DUT-OMRON.

Method	Input Size	Params(M)	FLOPs(G)	FPS	DUTS-TE				DUT-OMRON			
					*MAE*	$F_{\beta }^{max}$	$S_{m}$	*E*_*m*_	*MAE*	$F_{\beta }^{max}$	$S_{m}$	*E*_*m*_
CNN-Based Methods												
DFI	224 × 224	29.61	11.31	102	0.039	0.896	0.887	0.912	0.055	0.818	0.839	0.865
MINet-R	320 × 320	162.38	87.10	62	0.037	0.884	0.884	0.917	0.056	0.831	0.833	0.860
F3Net	352 × 352	25.54	16.48	167	0.035	0.905	0.888	0.920	0.053	0.841	0.838	0.864
LDF	352 × 352	25.15	15.57	177	0.034	0.905	0.892	0.925	0.052	0.835	0.839	0.865
GateNet-X	384 × 384	128.63	162.13	130	0.035	0.908	0.897	0.916	0.051	0.847	0.849	0.865
CAGNet-L	480 × 480	–	–	–	0.029	0.898	0.897	0.939	0.047	0.818	0.845	0.882
MENet	354 × 354	–	–	–	0.028	0.918	0.905	0.938	0.045	0.845	0.850	0.871
Transformer-Based Methods												
VST	224 × 224	44.48	23.18	70	0.037	0.895	0.896	0.919	0.058	0.836	0.850	0.871
ICON-S	384 × 384	92.15	52.80	69	0.025	0.924	0.917	**0.954**	0.043	0.862	0.869	0.900
SelfReformer	224 × 224	90.70	12.83	62	0.027	0.920	0.911	0.943	0.043	0.853	0.861	0.884
BBRF	352 × 352	74.00	67.02	62	0.025	0.911	0.909	0.949	0.044	0.839	0.861	0.896
SAM	512 × 512	89.94	103.17	46	0.030	0.921	0.909	0.937	0.044	0.865	0.869	0.896
MDSAM	384 × 384	100.21	66.23	56	0.025	0.933	0.919	0.950	0.040	0.878	0.876	0.908
**WMW-SAM**	384 × 384	95.52	63.23	62	**0.024**	**0.934**	**0.922**	0.952	**0.038**	**0.885**	**0.880**	**0.911**

The best and second-best results are marked in bold and underline, respectively.

**Table 2:** Quantitative comparison of our method with other methods on HKU-IS, ECSSD, and PASCAL-S.

Method	HKU-IS				ECSSD				PASCAL-S			
	*MAE*	$F_{\beta }^{max}$	$S_{m}$	*E*_*m*_	*MAE*	$F_{\beta }^{max}$	$S_{m}$	*E*_*m*_	*MAE*	$F_{\beta }^{max}$	$S_{m}$	*E*_*m*_
CNN-Based Methods												
DFI	0.031	0.934	0.920	0.951	0.035	0.949	0.927	0.924	0.065	0.885	0.857	0.861
MINet-R	0.029	0.942	0.919	0.952	0.033	0.954	0.925	0.950	0.064	0.881	0.856	0.896
F3Net	0.028	0.943	0.917	0.952	0.033	0.957	0.924	0.948	0.061	0.892	0.861	0.898
LDF	0.028	0.943	0.919	0.953	0.034	0.956	0.924	0.948	0.060	0.887	0.863	0.903
GateNet-X	0.029	0.946	0.925	0.947	0.035	0.957	0.929	0.944	0.064	0.892	0.865	0.895
CAGNet-L	0.024	0.940	0.923	0.961	0.026	0.950	0.930	0.959	0.063	0.878	0.870	0.917
MENet	0.023	0.951	0.927	0.960	**0.021**	0.957	0.928	0.951	0.053	0.897	0.872	0.910
Transformer-Based Methods												
VST	0.029	0.946	0.928	0.952	0.033	0.954	0.932	0.951	0.061	0.882	0.872	0.902
ICON-S	0.022	0.954	0.935	0.968	0.023	0.962	0.914	0.968	**0.048**	0.903	**0.885**	**0.924**
SelfReformer	0.024	0.949	0.931	0.960	0.027	0.959	0.936	0.957	0.051	0.902	0.881	0.919
BBRF	**0.020**	0.949	0.932	**0.969**	0.022	0.961	0.939	**0.969**	0.049	0.887	0.878	0.923
SAM	0.022	0.956	0.935	0.965	0.025	0.968	0.944	0.964	0.061	0.897	0.866	0.902
MDSAM	**0.020**	0.960	**0.940**	**0.969**	0.023	0.971	0.946	0.965	0.052	0.908	0.880	0.918
**WMW-SAM**	**0.020**	**0.961**	**0.940**	0.968	0.022	**0.973**	**0.947**	0.968	0.052	**0.912**	0.882	0.918

The best and second-best results are marked in bold and underline, respectively.

To evaluate the stability of our WMW-SAM, we repeat the experiments three times with different random seeds (42, 43, 44) on DUTS-TE and DUT-OMRON. Table 3 reports the means and standard deviations of *MAE*, $F_{\beta }^{max}$, $S_{m}$, and *E*_*m*_. The small standard deviations across the three runs demonstrate the robustness and reproducibility of our proposed method.

**Table 3:** Repeated-run results (mean ± std) of our method on DUTS-TE and DUT-OMRON.

Dataset	*MAE*	$F_{\beta }^{max}$	$S_{m}$	*E*_*m*_
DUTS-TE	0.024 ± 0.001	0.935 ± 0.002	0.922 ± 0.001	0.952 ± 0.001
DUT-OMRON	0.038 ± 0.001	0.884 ± 0.002	0.880 ± 0.001	0.911 ± 0.001

**Fig 7:**
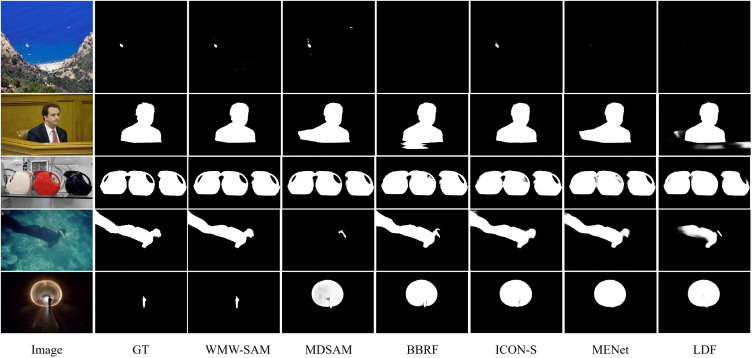
Visual comparison of saliency maps with our WMW-SAM and other methods.

**Qualitative Evaluation.** Fig 7 shows a visual comparison of our WMW-SAM with other approaches. Our method produces more accurate saliency maps in challenging scenes, such as small objects (row 1), large objects (row 2), multiple objects (row 3), low-contrast environments (row 4), and backlit scenes (row 5). These qualitative results indicate the strong performance and robustness achieved by our method.

Furthermore, we note recent advancements in SOD, including heavy architectures such as DualGazeNet [56] and BiRefNet [57], diffusion-guided generation paradigms like DGSSM [58], and highly efficient designs like CPDR [59]. While models with massive parameter capacities or iterative generation processes achieve higher accuracy, they often come with substantial computational costs. In contrast, our WMW-SAM achieves an effective balance between accuracy and efficiency. By introducing a few trainable parameters, our approach achieves superior performance over CPDR and approaches the accuracy of heavier architectures, while maintaining an inference speed of 62 FPS.

### 4.3. Ablation studies

**Fig 8:**
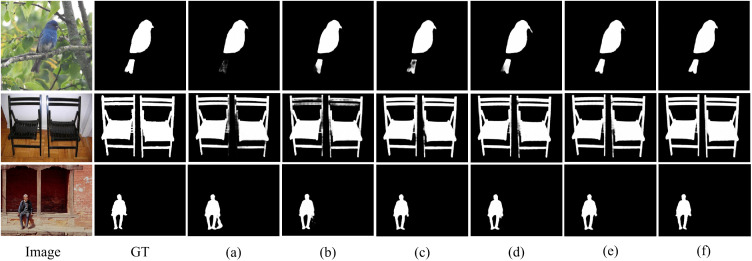
Visual comparisons for showing the benefits of the proposed modules. The image labels (a) to (f) correspond to model configurations in Table 4.

We perform ablation studies to evaluate each module in WMW-SAM. Table 4 shows the results on DUTS-TE and ECSSD. We use the frozen SAM with an LMSA adapter as the baseline. Based on this baseline, we add our WMSA, then MFCM, and finally the wavelet-enhanced DEM. Fig 8 provides a visual comparison of these configurations. All experiments in this section use a resolution of 384 × 384.

**Table 4:** Ablation studies of the proposed modules. MFCM* indicates using simple concatenation for fusion. DEM* indicates the removal of WA. The best results are marked in bold.

Method	DUTS-TE			ECSSD		
	*MAE*	$F_{\beta }^{max}$	$S_{m}$	*MAE*	$F_{\beta }^{max}$	$S_{m}$
(a)SAM+LMSA (Baseline)	0.027	0.926	0.916	0.025	0.968	0.944
(b)SAM+WMSA	0.026	0.927	0.918	0.024	0.969	0.945
(c)SAM+WMSA+MFCM*	0.026	0.928	0.918	0.023	0.970	0.945
(d)SAM+WMSA+MFCM	0.025	0.931	0.919	0.022	0.971	0.946
(e)SAM+WMSA+MFCM+DEM*	0.025	0.932	0.920	**0.021**	0.972	0.946
(f)SAM+WMSA+MFCM+DEM	**0.024**	**0.934**	**0.922**	0.022	**0.973**	**0.947**

**Effectiveness of WMSA.** We verify the effectiveness of WMSA by replacing LMSA with WMSA in the baseline model. As shown in Table 4, this change improves performance on all metrics. Furthermore, the visual comparisons in Fig 8 reveal that the baseline performs poorly on objects of different scales. With the introduction of WMSA, the model generates more accurate segmentation masks for both small and large objects. This shows that WMSA makes the model more adaptable to objects of various sizes.

**Effectiveness of MFCM.** Table 4 shows the effect of MFCM. The performance gain from row 2 to row 3 is marginal. This is because simple concatenation fails to effectively filter or fuse multi-level features and may even introduce noise, which degrades overall performance. With the integration of the MFCM, the accuracy improves more pronouncedly. As shown in Fig 8, simple concatenation still produces blurred edges, while MFCM helps the model capture object shapes and boundaries more completely. This leads to better detection results.

**Effectiveness of DEM.** Rows 5 and 6 of Table 4 give the quantitative results for the DEM and its WA. A basic DEM without WA brings only a small gain, which suggests that spatial-domain operation struggles to recover fine details. When WA is added, performance improves more clearly. This is because WA works in the frequency-domain and helps the model focus on high-frequency signals related to object boundaries. As a result, the model captures richer structural information. Fig 8 shows that WA enables the model to create sharper boundaries and finer textures, and thus yields improved saliency maps.

## 5. Conclusion

In this paper, we propose WMW-SAM, a novel framework that adapts SAM to the SOD task while preserving its pre-trained knowledge. To address the inherent limitations of previous adaptation strategies, we introduce three core components. First, WMSA employs learnable weights across multiple receptive fields. This design enables the encoder to handle salient objects of diverse sizes with few extra parameters, thus reducing incomplete predictions. Second, MFCM utilizes cascaded top-down cross-attention to enable effective interaction that connects high-level semantics with low-level details. This deep interaction helps to generate sharper object boundaries. Third, DEM leverages DWT to explicitly isolate and enhance high-frequency sub-bands in the frequency-domain. This operation recovers fine textures and edge information that tend to be discarded in spatial-domain processing. Extensive experiments on benchmark datasets confirm that our method yields more accurate saliency maps in complex scenes.
